# Translation, cross-cultural adaptation and validation of the Weight Management Questionnaire in the Brazilian population: a cross-sectional study

**DOI:** 10.1590/1516-3180.2024.0149.R2.10042025

**Published:** 2025-07-14

**Authors:** Brígida Rodrigues da Costa, Almir Vieira Dibai-Filho, Aldair Darlan Santos-de-Araújo, Fábio Henrique Ferreira Pereira, Daniela Bassi-Dibai, Adriana Sousa Rêgo

**Affiliations:** I Programa de Pós-Graduação em Meio Ambiente, Universidade Ceuma (Ceuma), São Luís (MA), Brazil.; II Programa de Pós-Graduação em Educação Física, Universidade Federal do Maranhão (UFMA), São Luís (MA), Brazil.; III Programa de Pós-Graduação em Fisioterapia, Universidade Federal de São Carlos (UFSCar), São Carlos (SP), Brazil.; IV Programa de Pós-Graduação em Meio Ambiente, Universidade Ceuma (Ceuma), São Luís (MA), Brazil.; V Programa de Pós-Graduação em Meio Ambiente, Universidade Ceuma (Ceuma), São Luís (MA), Brazil.; VI Programa de Pós-Graduação em Meio Ambiente, Universidade Ceuma (Ceuma), São Luís (MA), Brazil.

**Keywords:** Surveys and questionnaires, Obesity, Sedentary behavior, Weight management questionnaire, Lifestyle, Diet, Exercise

## Abstract

**BACKGROUND:**

The prevalence of obesity has increased considerably worldwide, and it has become an important risk factor for chronic non-communicable diseases.

**BJECTIVE:**

To translate, cross-culturally adapt, and validate the Weight Management Questionnaire (WMQ) to evaluate weight control practices for health monitoring and intervention in Brazil.

**DESIGN AND SETTING:**

This was a quantitative cross-sectional study.

**METHODS:**

Obese and physically active lean individuals aged 18−59 years were included in this study. The tool used as an adaptation was the WMQ. The cross-cultural adaptation was conducted in five phases. For structural validity, confirmatory factor analysis (CFA) was used to verify the internal structure considering two domains: diet and physical activity. To determine construct validity, a comparison was performed between different groups (lean versus obese). Reliability was assessed using the intraclass correlation coefficient (ICC).

**RESULTS:**

The short version of the WMQ after CFA presented eight items. Thus, the structure with two domains presented adequate fit indices: chi-square/degree of freedom = 1.66, comparative fit index = 0.996, Tucker-Lewis index = 0.994, root mean square error of approximation = 0.057, and standardized root mean square residual = 0.047. For construct validity, a significant difference was observed between the groups (P < 0.05) in both domains. We observed adequate reliability for both domains (ICC ≥ 0.854).

**CONCLUSIONS:**

The WMQ can be used for the Brazilian population, as it is reliable and has adequate internal structure, supporting its use in future research.

## INTRODUCTION

 The World Health Organization (WHO) defines obesity as a chronic condition determined by excessive fat accumulation that leads to negative health.^1^The prevalence of obesity has increased considerably worldwide in the last decades, which has resulted in an increase in lifestyle-related diseases. Obesity is an important risk factor for non-communicable diseases (NCD), such as diabetes, hypertension, and cardiovascular diseases, among others.^2^

 A sedentary lifestyle has become one of the most important risk factors for the development of NCD. Although there is sufficient scientific evidence to confirm the benefits of regular physical activity (PA), currently 31.1% of the global adult population does not meet the minimum PA recommendations (≥ 150 min of moderate or vigorous intensity activity per week).^3^Adherence to an unhealthy lifestyle may cause obesity and, in consequence, NCD. According to the WHO, lifestyle is defined as a set of behaviors and habits that are influenced by the process of socialization; they comprise the consumption of substances such as tobacco, alcohol, coffee, tea, dietary habits, and physical exercise.^4^

 Epidemiologically, in developing countries, obesity mainly affects middle-aged adults, whereas in developed countries, it affects both sexes at the same proportion and people of all age ranges.^5^Overweight and obesity may reach levels of 89% and 85% in men and women, respectively, by 2030. This will cause an increase in the prevalence of obesity-related coronary cardiac disease by 97%, cancer by 61%, and type 2 diabetes by 21%. Therefore, health costs increase considerably, overloading the public health system.^5^

 Currently, in Brazil, various instruments analyze obesity and physical activity in isolation in different populations. An example of this is the Lipedema Screening Questionnaire that assesses knowledge about this clinical condition in women.^6^Another tool committed to investigating the level of physical activity in Brazilians in the last 12 months is the Baecke Habitual Physical Activity Questionnaire (BHPAQ).^7^

 Advice on lifestyle changes, such as nutritional education on calorie deficits and regular exercise, is the cornerstone of managing all lifestyle-related diseases. Understanding the determinants of non-adherence to advice on lifestyle changes may help physicians plan and develop interventions focused on assisting patients in achieving long-term healthy weight loss.^8^

 In this context, the Weight Management Questionnaire (WMQ) was created and validated in India with 14 items in a Likert scale format and divided into two domains: items 1 to 12 are related to the diet domain, and items 13 and 14 are related to the physical activity domain. The questionnaire showed satisfactory structural validity and adequate internal consistency.^8^ To the best of our knowledge, only the original version of the WMQ has been published,^8^ thus our study is the first cross-cultural adaptation. 

 The rationale for translating and adapting the WMQ is the need for a valid, reliable, and culturally appropriate instrument to assess eating habits and physical activity of Brazilian individuals. The hypothesis of this study was that there would be significant differences in the WMQ domain scores between the groups. Additionally, we expect a reliable two-dimensional instrument with adequate internal consistency. Given the scenario of validated instruments in Brazil and the importance of this tool, the need arose to translate, cross-culturally adapt, and validate the WMQ for Brazilian Portuguese by assessing eating habits and physical activity using a single tool in a succinct and objective way. To date, this questionnaire has not been validated in any country, and this is the first validation study of this tool. 

## METHODS

### Study design

 This quantitative cross-sectional study was conducted in São Luís (the capital of Maranhão, Northeast Brazil). São Luís is located on the coast of Maranhão and is officially part of the Brazilian Legal Amazon.^9^ All participants included in the study validated their participation by signing an informed consent form. This study was approved by the institutional ethics committee (report number: 2,853,570). Before validating the questionnaire in the Brazilian population, written consent was obtained from the author of the original manuscript (Dr. Piyush Ranjan). 

### Sample size

 For sample size, we adopted the guidelines of the Consensus-based Standards for the Selection of Health Measurement Instruments (COSMIN), which consider a sample size seven times the number of items of the instrument as long as the minimal sample size is 100 participants.^10^

 Obese individuals with a body mass index (BMI) > 30 kg/m^2^ and physically active lean individuals (BMI < 24.9 k/m^2^) aged 18−59 years, were included in this study. We excluded individuals who were unable to read and write Brazilian Portuguese, with cognitive or neurological alterations, or with any impairment in their ability to reply to the questionnaire. 

### Instruments

 The data were collected online (Google Forms, Mountain View, California). To characterize the sample, we evaluated sociodemographic and personal data using the BHPAQ, a self-applicable self-report instrument that evaluates physical activity over the last 12 months. The BHPAQ is composed of 16 items divided into three domains: occupational physical activity (items 1−8), free-time sports physical activity (items 9−12), and leisure nonsports physical activity (items 13−16). Each domain is considered separately to calculate the final score. The total score ranged from 1 to 5; the higher the score, the greater the habitual physical activity. The BHPAQ has been adapted and validated in the Brazilian Portuguese population.^7^

 The target tool of this study was the WMQ, an instrument that evaluates adherence to diet and exercises for weight management.^8^ The questionnaire has 14 items in two domains: 12 items on the "diet" domain and 2 items on the "physical activity" domain. The responses were based on a Likert scale ranging from 1 to 5 points. Originally, the values obtained were added to the scores for each domain. 

## TRANSLATION AND ADAPTATION

 Translation and adaptation into Brazilian Portuguese were performed in accordance with the Guidelines for the Process of Cross-Cultural Adaptation of Self-Report Measures,^11^ which are divided into five steps:

1. Translation: Two independent translators, with Brazilian Portuguese as their native language and fluency in English, translated the original version of the questionnaire into Brazilian Portuguese.
2. Synthesis of the translations: After discussions and reviews, both translators observed by one of the researchers synthesized the two translated versions of the questionnaire independently and produced a single version in a consensual way.
3. Back-translation: Two independent translators (with no technical background in the health area and blind to the original version of the questionnaire), both with English as their mother tongue and fluency in Portuguese, translated the Portuguese version of the questionnaire back to English.
4. Expert committee analysis: Six experts in the fields of nutrition, physical exercise, and health and wellness reviewed all translated and back-translated versions and corrected any discrepancies, creating a pre-final version of the WMQ.
5. Pre-final version: To test the pre-final version, the sample comprised of 34 randomly selected participants. Next, to check the instrument’s measurement properties, the final version of the transculturally adapted WMQ was administered to 203 individuals (101 obese and 102 physically active, lean individuals).

## DATA ANALYSIS

 In terms of descriptive analysis, quantitative variables are presented as means and standard deviations, and qualitative variables are presented as numbers and percentages. Comparisons between the lean and obese groups were made using an independent t-test or chi-square test, according to the normality of the data. 

 For test-retest reliability analysis, we used a subsample of 34 participants. The individuals answered the WMQ at two time points, with an interval of seven days between evaluations.^12^ We used the intraclass correlation coefficient (ICC), and a value equal to or higher than 0.75 was considered as the acceptability cut-off.^13^-^15^ In addition, the standard error of measurement (SEM) and minimal detectable difference (MDD) were calculated.^12^

 For structural validity, confirmatory factor analysis (CFA) was performed using R Studio (Boston) with the packages lavaan and semPlot. The WMQ is scored on a Likert scale (ordinal data). Therefore, CFA was performed by implementing a polychoric matrix and the extraction method using robust diagonally weighted least squares. The model fit was assessed using the following indices: root mean square error of approximation (RMSEA) with a confidence interval (CI) of 90%, comparative fit index (CFI), Tucker-Lewis index (TLI), standardized root mean square residual (SRMR), and chi-square/degrees of freedom (DF). 

 Values higher than 0.90 were considered appropriate for CFI and TLI, and values lower than 0.08 were considered appropriate for RMSEA and SRMR. Values below 3 were considered appropriate for the interpretation of chi-square/DF.^13^ In CFA, factor loadings equal to or higher than 0.40 were considered appropriate for the domain. For WMQ refinement, modification indices > 10 were used to identify redundant items and items with lower factor loadings in every paired analysis were excluded.^14^

 To determine construct validity, a comparison between recognizably distinct groups (lean versus obese) was performed using a t-test for independent samples, with a significance level of 5%.^10^ The effect size was calculated based on Cohen’s d, according to the website https://www.psychometrica.de/effect_size.html, with the following interpretations of the d value: 0.2 (weak), 0.5(moderate) and > 0.8 (large effect size). Descriptive analysis, construct validity, and reliability were analyzed using SPSS software(version 17.0, Chicago). 

## RESULTS

### Translation and cross-cultural adaptation

 After translating the WMQ, we adapted the food items mentioned in the questionnaire to the Brazilian context. These adaptations were performed for items 3, 4, 5, 6, and 9, as shown in **Table 1**. There was 100% understanding of the items in the prefinal version of the WMQ. 

**Table 1:** Adaptation of food items of the Weight Management Questionnaire (WMQ)

**Translated version**	**Adapted version**
Item 3: laddu, barfi, jalebi, kulfi, chocolate, halwa, arroz doce.	Item 3: brigadeiro, cocada, doce de leite, goiabada, paçoca, pudim.
Item 4: puri, parathas, kachori, tikki, bhature, pakoras, samosas.	Item 4: pastel, batata frita, torresmo, salgados, alimentos fritos.
Item 5: namkeen, bhujia, pickles, chutney, papad.	Item 5: fast food, macarrão instantâneo, pipoca industrializada, salgadinhos industrializados.
Item 6: coalhada, lassi.	Item 6: suco, achocolatado.
Item 9: gordura de carneiro.	Item 9: manteiga, queijo.

### Structural validity

 CFA was employed to check the internal structure, considering two domains according to the original proposal of the questionnaire (Model 1): the diet domain (items 1 to 12) and the physical activity domain (items 13 and 14). The proposed model showed an inappropriate internal structure. As the internal structure of the proposed model was considered inappropriate, we excluded items with factor loadings below 0.40, including items 1 (0.19), 2 (0.33), 6 (0.29), and 9 (0.26). Subsequently, we applied CFA again with the exclusion of these four items from the diet domain (Model 2), and the values of the fit indices remained inappropriate (except for CFI): chi-square/DF = 9.77, CFI = 0.903, TLI = 0.871, RMSEA (90% CI) = 0.208 (0.188 to 0.229), and SRMR = 0.153, as reported in **Table 2**. 

**Table 2:** Fit indices after confirmatory factor analysis of the Weight Management Questionnaire (WMQ)

**Fit indices**	**Model 1**	**Model 2**
Chi-square/DF	7.35	9.77
CFI	0.846	0.903*
TLI	0.815	0.871
RMSEA (90% CI)	0.177 (0.164 to 0.191)	0.208 (0.188 to 0.229)
SRMR	0.15	0.153

* RMSEA, root mean square error of approximation; CFI, comparative fit index; TLI, Tucker-Lewis index; SRMR, standardized root mean square residual; DF, degrees of freedom. * Adequate fit index.

 With Model 2, the issues of the model were investigated using modification indices, and high correlations were identified among the instrument items, as indicated in **Table 3**, which resulted in the exclusion of items 7 and 8 (lowest factor loading in the analysis paired with modification indices). Following the exclusion of items 7 and 8, CFA was employed again, considering items 3, 4, 5, 10, 11, and 12 for the diet domain, and items 13 and 14 for the physical activity domain (Model 3), producing appropriate fit indices: chi-square/DF = 1.66, CFI = 0.996, TLI = 0.994, RMSEA (90% CI) = 0.057 (0.016 to 0.092), and SRMR = 0.047. 

**Table 3:** Modification items (MI) and items excluded from Weight Management Questionnaire (WMQ)

**Item**	**Description**	**Factor loading**	**MI**	**Item excluded**
Item 7	How often do you eat fruit and salad?	0.68	228.336	Item 8
Item 8	How often do you eat vegetables and greens?	0.63		
Item 7	How often do you eat fruit and salad?	0.68	22.432	Item 7
Item 13	How many times do you exercise in a week?	0.95		

**Figure 1:**
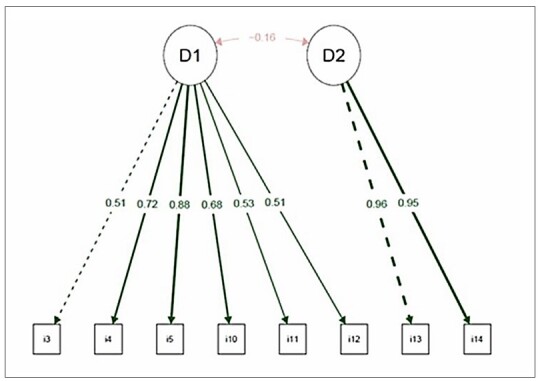
Path diagram showing the relationship between WMQ domains and items with their respective factor loadings.

**Figure 1** shows the relationship between the WMQ domains and the items with appropriate factor loadings (> 0.4). The diet (D1) and physical activity (D2) domains were negatively correlated; that is, they were inversely proportional. Therefore, the scores of the short version of the WMQ should be determined by domain, with diet domain scores from 6 to 30, and physical activity domain scores from 2 to 10. For interpretation, the higher the score in the diet domain, the better the individual’s eating habits; the lower the score in the physical activity domain, the more active the individual. The Brazilian version of the WMQ can be accessed through the website https://questionariosbrasil.blogspot.com/. 

### Data descriptive analysis

Table 4 shows sample description, in addition to comparisons between the lean and obese groups. Significant differences were observed for weight, BMI, BHPAQ sports and leisure domain, and education level. 

**Table 4:** Comparison among the variables of sample characterization of the active lean group and obese group

**Quantitative variables**	**Active lean group (n = 102)**		**Obese group (n = 101)**		**P value**
	**Mean**	**Standard deviation**	**Mean**	**Standard deviation**	
Age (years)	29.35	7.93	30.27	7.39	0.392
Weight (kg)	63.89	9.5	95.16	16.34	0.001^a^
Height (m)	1.68	0.09	1.68	0.1	0.929
BMI (kg/m^2^)	22.45	1.64	33.56	3.77	0.001^a^
BHPAQ-O (score, 1−5)	2.56	0.64	2.56	0.61	0.992
BHPAQ-S (score, 1−5)	2.96	0.68	2.15	0.82	0.001^a^
BHPAQ-L (score, 1−5)	2.66	0.59	2.36	0.67	0.001^a^
**Qualitative variables**	**n**	**%**	**n**	**%**	**P value**
Sex	102		101		0.529
*Female*	56	54.9	50	49.5	
*Male*	46	45.06	51	40.59	
Education					**0.038^b^**
*Incomplete secondary education*	1	0.98	1	0.99	
*Completed secondary education*	11	10.78	25	24.75	
*Incomplete higher education*	23	22.548	13	12.87	
*Completed higher education*	38	37.25	34	33.66	
*Incomplete post-graduation*	12	11.76	5	4.95	
*Completed post-graduation*	17	16.66	23	22.77	
Marital status					0.242
*Single*	84	82.35	72	71.28	
*Married*	13	12.74	24	23.76	
*Divorced*	4	3.92	4	3.96	
*Widower*	1	0.98	1	0.99	

BHPAQ-O: occupational domain of the Baecke Habitual Physical Activity Questionnaire; BHPAQ-S: sports domain of the Baecke Habitual Physical Activity Questionnaire; BHPAQ-L: leisure domain of the Baecke Habitual Physical Activity Questionnaire. ^a^ Significant difference (independent t-test, P < 0.05); ^b^ Significant difference (chi-square test, P < 0.05).

### Construct validity

 Construct validity was conducted by comparing two recognizably distinct groups: physically active lean individuals (BMI 18.524.9 kg/m^2^) versus obese individuals (BMI > 30 kg/m^2^). As **Table 5** shows, a significant difference was observed between the groups (P < 0.05), indicating that the WMQ construct was valid. 

**Table 5:** Comparison between the scores of the domains of the Weight Management Questionnaire (WMQ)

**WMQ domains**	**Lean group**	**Obese group**	**p value**	**Cohen’s d**
Diet (6−30)	20.78	18.49	0.005*	0.442
	(4.9)	(5.44)		(0.039, 0.923)
Physical activity (2−10)	3.37	7.27	< 0.001*	1.704
	(1.14)	(3.03)		(1.149, 2.259)

* Significant difference (P < 0.05, independent t-test); Cohen’s d interpretation: 0.2 (weak), 0.5 (moderate) and > 0.8 (large effect sizes).

### Test-retest reliability

 In terms of the diet domain, appropriate test-retest reliability was observed in active lean (ICC = 0.977) and obese (CCI = 0.854) participants. Similarly, in the physical activity domain, satisfactory test-retest reliability was observed for the active lean (ICC = 0.940) and obese (ICC = 0.924) participants. Further details are presented in Table 6.

**Table 6:** Reliability of the Weight Management Questionnaire (WMQ)

**Parameters**	**Lean Values(n = 34)**	**Obese Values(n = 34)**
Diet domain		
Test, mean (standard deviation)	21.32 (5.34)	17.76 (4.17)
Retest, mean (standard deviation)	20.88 (5.55)	18.02 (3.67)
ICC (95% CI)	0.977 (0.954, 0.988)	0.854 (0.707, 0.927)
SEM, score (%)	0.83 (3.91%)	1.50 (8.37%)
MDD, score (%)	2.29 (10.85%)	4.15 (23.31%)
Physical activity domain		
Test, mean (standard deviation)	3.32 (0.97)	7.61 (2.75)
Retest, mean (standard deviation)	3.41 (0.92)	6.73 (3.69)
ICC (95% CI)	0.94 (0.88, 0.97)	0.924 (0.847, 0.962)
SEM, score (%)	0.23 (6.88%)	0.89 (12.38%)
MDD, score (%)	0.64 (19.07%)	2.46 (34.32%)

ICC, intraclass correlation coefficient; CI, confidence interval; SEM, standard error of measurement; MDD, minimal detectable difference.

## DISCUSSION

 This study aimed to translate, transculturally adapt, and validate the WMQ in Brazilian Portuguese. After translation, synthesis of translations, back-translation, expert committee analysis, and a pre-final version test, the instrument was applied to a sample of 203 participants. The original questionnaire was created and validated for obese individuals with nonalcoholic fatty liver disease; however, it is worth mentioning that this Brazilian adaptation was made only for obese individuals because the questionnaire has generic characteristics. 

 For the short version of the WMQ for the Brazilian population, a two-dimensional tool was proposed with two domains: a diet domain with a score between 6 and 30, and a physical activity domain with a score between 2 and 10, presenting a valid internal structure with eight items after the execution of the structural validity. To interpret data from the short version, the higher the diet domain score, the better the eating habits, and the lower the physical activity domain score, the more active the individual, as the questionnaire domains are inversely proportional. 

 The questionnaire revealed a significant difference between the two distinct groups (obese versus active lean), thus presenting a valid construct. Regarding test-retest reliability, adequate reliability was observed in the active lean (ICC = 0.977) and obese (ICC = 0.854) participants. Similarly, for the physical activity domain, satisfactory test-retest reliability was observed for the active lean (ICC = 0.94) and obese (ICC = 0.924) participants. 

 It is important to highlight the similarities and differences between the original and our proposed versions of the WMQ. The original version used exploratory factor analysis, but did not present the model fit indices for a more adequate understanding of the analysis. Also, the authors did not clearly describe the method used to identify the number of domains of the instrument, nor the factor loadings of the relationship between domain and items.^8^ Our study used CFA considering the two-dimensional structure, however, some items were redundant. The shortened version proposed here presented adequate fit indices (chi-square/DF < 3, CFI and TLI > 0.9, RMSEA and SRMR < 0.08). In this sense, it is important that future studies in different cultures compare different structures to determine the most appropriate structure. 

 In addition to the factor analysis, the original version of the WMQ showed adequate internal consistency (Cronbach’s alpha = 0.94). Our study did not use Cronbach’s alpha because of the small number of items in the physical activity domain (only two items). However, the CFA we used was a much more robust analysis of the relationships between items and domains. In addition, our study identified adequate reliability in both domains; the original study did not evaluate reliability using ICC.^8^

 Duarte et al.^16^ reported the importance of translation, cultural adaptation, and validation of questionnaires in the health field, stressing that the adaptation of a questionnaire for use in a new culturally different population, when it is valid for the population studied, is the best and most appropriate way of guaranteeing a reliable and reproducible instrument, corroborating our results for the WMQ. 

 Concerning questionnaires similar to the constructs tested here, one study proposed the development of a new Portuguese version of the Sociocultural Attitudes Toward Appearance Questionnaire-4 (SATAQ-4),^17^ culturally adapting it for use in different contexts of Brazilian Portuguese and evaluating its validity and reliability when applied to a sample of university students. The SATAQ-4 showed appropriate structural validity (CFI = 0.98, TLI = 0.98, RMSEA = 0.08), which was consistent with the WMQ values (CFI = 0.99, TLI = 0.99, RMSEA = 0.057). 

 The Exercise Adherence Rating Scale (EARS) is a tool designed to evaluate adherence to prescribed home physical activity, exclusively intended for individuals with chronic back pain.^18^ The EARS-Br scale was translated, adapted, and validated to Brazilian Portuguese and presented acceptable internal consistency (α = 0.88) and excellent reliability (ICC = 0.91).^19^ The WMQ reliability was assessed based on a test-retest model, using the ICC, and it was considered appropriate and reliable, similarly to EARS-Br. 

 The consumption of ultra-processed food has grown considerably in Brazil; as a result, the prevalence of obesity has increased across all age ranges. For this reason, a study was conducted to test the validity and reliability of the Portuguese version of the Comprehensive Feeding Practices Questionnaire (CFPQ), which was translated, adapted, and applied to a sample of parents of preschool Brazilian children. The modified version of the CFPQ demonstrated significant internal reliability and appropriate validity in the population studied.^20^

 Food literacy refers to the knowledge and competencies related to healthy food choices. Based on that, the process of transcultural adaptation and content validation of the Short Food Literacy Questionnaire (SFLQ) was performed for the Brazilian population.^21^ The SFLQ-Br showed to be reliable, valid and stable, which assures that the instrument can be deemed useful to evaluate the food literacy of Brazilian adults. 

 The WMQ construct validity was satisfactory when comparing obese versus active lean individuals, as it produced a significant difference. The internal consistency indicated an appropriate value and the test-retest reliability was acceptable for the diet and physical activity domains. 

 The present study has several limitations. One of the main limitations of this study was that we did not perform an analysis of criterion validity because there was no pre-existing validated instrument with which to directly compare the WMQ. Although we performed CFA to assess structural validity, we did not conduct exploratory factor analysis, which could have provided further insight into the dimensionality of the WMQ. As our study had a cross-sectional design, it was not possible to assess responsiveness or identify the minimum clinically important differences. In addition, the database was established online and only in one region of Brazil. Therefore, emphasizing the importance of a country’s cultural plurality, we recommend that further studies apply this tool to different regions of the country to corroborate the reproducibility of our study. 

 Regarding the clinical implications, this is the first study to translate, adapt, and validate the Indian version of the WMQ into Brazilian Portuguese, and we have shown it can be considered a reliable, valid, and useful tool in both clinical and scientific contexts. The direct use of the WMQ allows for the comprehensive assessment of eating habits and physical activity levels. The questionnaire consists of domains related to diet and exercise, enabling a detailed evaluation of lifestyle behaviors. In practice, the instrument can provide valuable data for healthcare professionals who, by using it, will be able to identify patterns and specific areas that will guide more personalized interventions, supporting more effective strategies for health promotion and control of diseases arising from sedentary lifestyles and improper eating habits. 

## CONCLUSION

 The WMQ can be used in the Brazilian population as a reliable tool with an adequate internal structure and constructs, supporting its use in future research applications. 
